# Supplementary material for: HIVprotI: an integrated web based platform for prediction and design of HIV proteins inhibitors
Source: J Cheminform. 2018 Mar 9;10:12. doi: 10.1186/s13321-018-0266-y (PMC5845081; doi:10.1186/s13321-018-0266-y)
Supplement: Supplementary file 2 — Additional file 2. Source code of HIVProtI web server. [file 13321_2018_266_MOESM2_ESM.zip › HIVprotI_Source-code/hivprotI/weka-3-6-6/doc/weka/datagenerators/clusterers/package-summary.html]

weka.datagenerators.clusterers


---


|  |  |  |
| --- | --- | --- |
| **Overview** \| **Package** \| Class \| **Tree** \| **Deprecated** \| **Index** \| **Help** \| **Weka's home** | |  |
| **PREV PACKAGE**   **NEXT PACKAGE** | **FRAMES**    **NO FRAMES**     **All Classes** |  |


---

## Package weka.datagenerators.clusterers

| **Class Summary** | |
| --- | --- |
| **BIRCHCluster** | Cluster data generator designed for the BIRCH System    Dataset is generated with instances in K clusters.  Instances are 2-d data points.  Each cluster is characterized by the number of data points in itits radius and its center. |
| **SubspaceCluster** | A data generator that produces data points in hyperrectangular subspace clusters. |
| **SubspaceClusterDefinition** | A single cluster for the SubspaceCluster datagenerator   Valid options are: |

---


|  |  |  |
| --- | --- | --- |
| **Overview** \| **Package** \| Class \| **Tree** \| **Deprecated** \| **Index** \| **Help** \| **Weka's home** | |  |
| **PREV PACKAGE**   **NEXT PACKAGE** | **FRAMES**    **NO FRAMES**     **All Classes** |  |


---
